# Outcomes of Heavy Silicone Oil (Densiron) compared to Silicone Oil in primary rhegmatogenous retinal detachment: a multivariable regression model

**DOI:** 10.1186/s40942-022-00413-0

**Published:** 2022-09-03

**Authors:** George Moussa, Maria Tadros, Soon Wai Ch’ng, Ash Sharma, Kim Son Lett, Arijit Mitra, Ajai K. Tyagi, Walter Andreatta

**Affiliations:** 1 Birmingham and Midland Eye Centre and Academic Unit of Ophthalmology, University of Birmingham, Birmingham, UK; 2 Birmingham and Midland Eye Centre, Sandwell and West, Birmingham Hospitals NHS Trust, Dudley Road, Birmingham, B18 7QH UK; 3 Kantonsspital Winterthur, Brauerstrasse 15, 8400 Winterthur, Switzerland; 4 University of Zurich, Rämistrasse 71, 8006 Zurich, Switzerland

**Keywords:** Retinal detachment, Silicone oil, Heavy oil, Heavy silicone oil, Densiron, Outcomes, Vitreoretinal, Retina, Glaucoma, Retinectomy

## Abstract

**Purpose:**

To measure the visual outcomes, proliferative vitreoretinopathy (PVR) and retinectomy rates following primary rhegmatogenous retinal detachment (RRD) repair, comparing silicone oil (SO) and heavy SO (Densiron).

**Methods:**

Retrospective, continuous comparative study from January 2017 to May 2021 of all primary RRD. Multivariable linear (logMAR gain) and binary-logistic (PVR-C and retinectomy rate) regression models to compare tamponade were performed. Covariates included age, gender, ocular co-morbidities, high myopia, macula-status, giant-retinal-tear (GRT), pre-op vision, PVR-C, oil type, perfluorocarbon-use, combined scleral buckle/vitrectomy, combined phaco-vitrectomy, 360-degrees-endolaser and oil duration. Cases with trauma or less than six-month follow-up were excluded.

**Results:**

A total of 259 primary RD were analysed. There were 179 SO patients and 80 Densiron patients that had six-month primary re-detachment in 18 (10.1%) and 8 (10.0%) respectively (p = 1.000). No difference in logMAR gain was detected between tamponade choice on multivariable linear regression. Subsequent glaucoma surgery was 5 (2.8%) and 4 (5.0%) for SO and Densiron patients respectively (p = 0.464). On multivariate binary-logistic regression we found no difference in development of PVR-C between oil tamponades. However, SO had significantly higher subsequent retinectomy rate compared to Densiron (odds ratio 15.3, 95% CI 1.9–125.5, p = 0.011). Duration of oil tamponade was not linked to differences in logMAR gain, PVR-C formation or increased retinectomy rate.

**Conclusions:**

We report no difference in primary anatomical success, number of further RRD surgeries, subsequent glaucoma surgery, visual outcomes, PVR-C between both tamponades on multivariable models. Densiron oil was found to be more retinectomy sparing relative to SO.

## Introduction

While gas tamponades are often utilised in the treatment of rhegmatogenous retinal detachments (RRD), specific characteristics of RRD can lead to the clinical decision to use silicone oil (SO) tamponade agents. These include, but are not limited to, factors such as their chronicity, the location and morphology of breaks, presence of proliferative vitreoretinopathy (PVR), and the likelihood of strict posturing by patients. However, as SOs are of lower density than water and have limited ability to support the inferior retina, tamponade agents with heavier-than-water properties, such as heavy SO (HSO) may be selected to support these areas of pathology [1]. Densiron®68 (Fluoron Co, Neu-Ulm, Germany) is a HSO that is a solution of 30.5% perfluorohexyloctane (F_6_H_8_) and 69.5% SO 5000cs [2]. It is an effective tamponade for inferior retinal detachments and particularly useful in patients that cannot perform prone posturing.

However, HSOs have their own hypothesised risk profiles. Heimann et al. postulated a foreign-body reaction to emulsification of droplets of HSO that may result in superior PVR-type membranes [3, 4].

 It is thought that dispersion, or emulsification of droplets, leads to a heightened inflammatory response concentrated superiorly above the main bubble, resulting in the formation of precipitates, fibrin, PVR and epiretinal membranes that can potentially increase the retinectomy rate [5].

However, it can be difficult to distinguish complications attributable to the tamponade agent from sequelae of complex retinal pathology, particularly when multiple different tamponade agents are included in analysis. Therefore, we conducted this retrospective study to primarily assess the visual outcomes following primary repair of RRD with SO and Densiron. Our secondary outcomes include the retinectomy, PVR and glaucoma surgery rate following SO and Densiron tamponade.

## Methods

A single centre, retrospective, continuous and comparative study, to analyse all patients that had primary RRD performed at the Birmingham and Midland Eye Centre (BMEC). The study period covers 4.5 consecutive years from January 2017 to May 2021.

### Inclusion/exclusion criteria and definitions

Primary RRDs repaired by pars plana vitrectomy (PPV) were selected to reduce confounding factors of prior re-detachments and risk adjusted for case complexity to allow more meaningful comparison between both SO and Densiron. Exclusion criteria include post-traumatic RRD and lack of follow up (due to patients being referred back to other peripheral hospital units).

Primary failure was defined as a detachment under oil or the decision for permanent oil tamponade. Patients that were awaiting ROSO, with stable examination findings were not defined as failure. This was particularly important due to increased waiting list pressures due to the COVID-19 pandemic would falsely raise the six-month failure statistics.

### Data collection

All the data were extracted from electronic patient records (EPR, Medisoft Ophthalmology, Medisoft Limited, Leeds, UK). Data collection included:

I. Baseline demographics and characteristics [age, gender, pre-operative lens status, laterality, the presence of high myopia (defined as greater than six dioptres of myopia), pre-operative visual acuity (VA), macula status and ocular co-morbidities (including macular degeneration, vein occlusion, corneal pathology, glaucoma,
II. Intra-operative and post-operative factors (choice of tamponade, post-operative lens status, use of perfluorocarbon liquid (PFCL), requirement of PVR peel and/or retinectomy, retinal detachment re-operation rate (excluding routine removal of SO), rate of subsequent PVR C and retinectomy in patients with and without initial PVR C and retinectomy, rate of SO, Densiron and gas tamponade if subsequent RRD surgery was required, rate of post-operative glaucoma surgery and epiretinal membrane (ERM) peel, and duration of SO/Densiron tamponade). Patients must have had a minimum of six-month follow up to be included.

### Surgical technique

All RRD surgery was performed with transconjunctival 23-gauge PPV and retinopexy undertaken with cryotherapy, endolaser, a combination of both with or without three-sixty barrier laser. Surgery could be combined with a scleral buckle, involve PVR peeling for PVR Grade C, retinectomy and the use of PFCL when necessary. The choice of tamponade was a clinical decision based on the operating surgeon, including number, location and morphology of retinal breaks [especially giant retinal tears (GRTs)], the presence of PVR Grade C, RRD location and chronicity. Patients with reduced ability to posture or with inferior retinal detachments were more likely to receive Densiron compared to SO in our unit. Our SO was supplied by FCI Ophthalmics (FCI S.A.S. – France Chirurgie Instrumentation, 20–22 rue Louis Armand, 75,015 Paris, France).

### Statistical analysis

All statistical analysis was performed using IBM SPSS Statistics for Windows, Version 28.0 (IBM Corp, Armonk NY). Statistical significance was defined as p < 0.05. Prior to analysis, continuous variables were assessed using the Shapiro–Wilk test and found not to be normally distributed. Hence, data are primarily reported as medians and interquartile ranges (IQRs) throughout. For univariate comparisons, Mann Whitney U test was used to compare two groups respectively (age, VA and duration of oil tamponade). Wilcoxon signed rank test was used for two-paired VA data. Fisher exact test and Chi-Squared test were used for nominal variables.

Due to significant differences in case complexity between cases, we undertook several multivariable regression analyses. To investigate our primary outcome of visual outcomes following primary RRD repair, we conducted a multivariate linear regression analysis on logMAR gain (pre-operative logMAR minus post-operative logMAR) on visual outcome including only pseudophakic patients (to reduce lens opacities and aphakic patients as confounders). As covariates, we included (i) baseline demographics characteristics: age, gender, high myopia, presence of ocular comorbidities (other than high myopia), macula status, presence of GRT, pre-operative visual acuity and presence of PVR C, (ii) intraoperative characteristics: tamponade choice (SO or Densiron), retinectomy performed, combined scleral buckle with PPV, 360 degrees endolaser retinopexy perfomed, combined phacovitrectomy performed and (iii) post-operative characteristics: duration of oil tamponade.

Best corrected VA was used and records in Snellen were converted to logMAR. Low VA, corresponding to count fingers (CF), hand movements (HM), perception of light (PL) and no PL (NPL) were substituted with 2.10, 2.40, 2.70 and 3.00 LogMAR, respectively, in keeping with previous publications from the national ophthalmology database group [6], using a tool by Moussa et al. [7] For our secondary outcomes, we also carried out multivariable binary logistic regression analyses with subsequent retinectomy rate and PVR C rate as dependent variables.

## Results

**Fig. 1:**
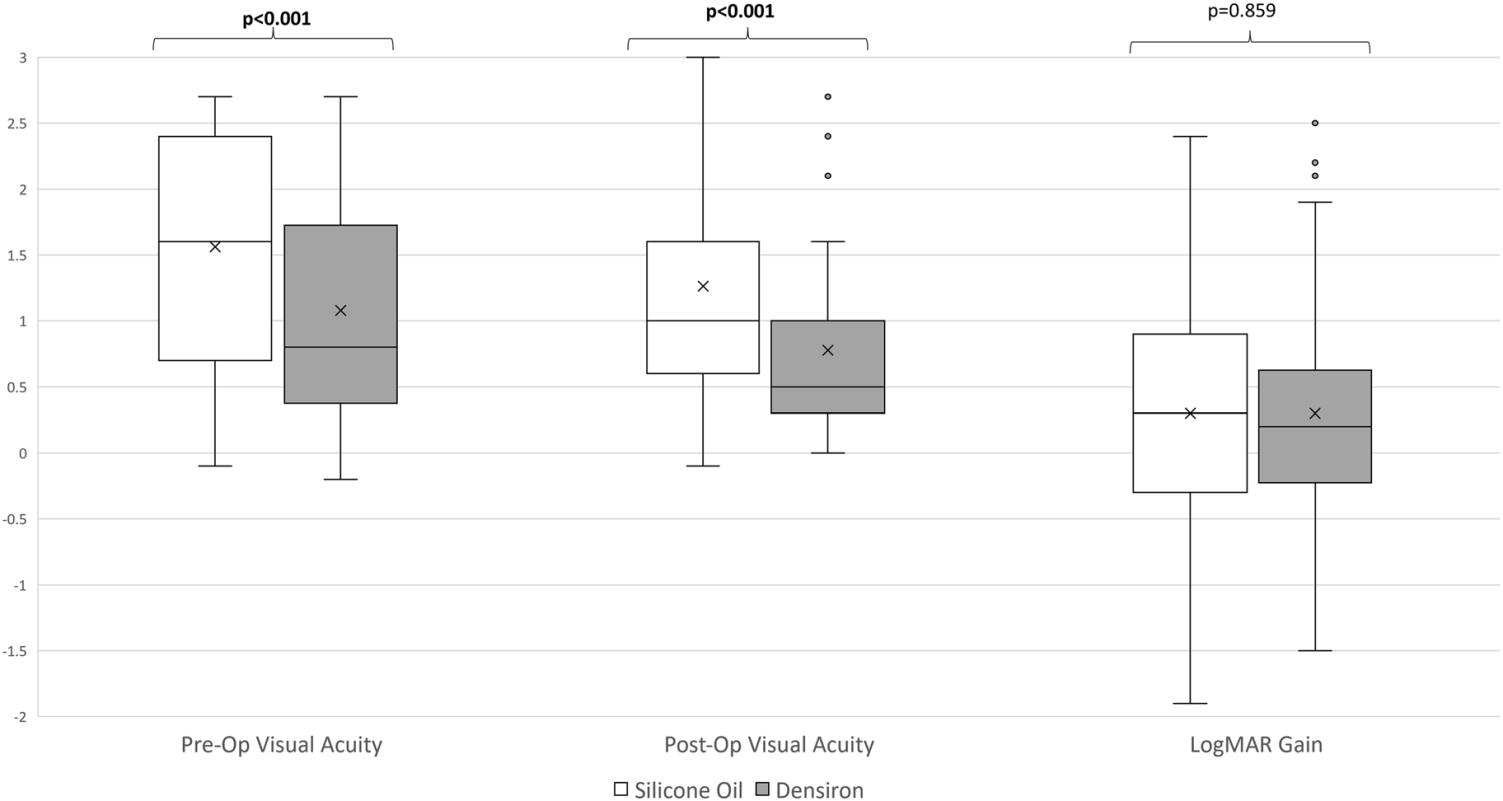
Box and Whisker Plot of Visual Acuity Baseline and Outcomes by Tamponade. Box and Whisker plot. ‘X’ denotes mean. *Mann Whitney U-test. Statistical significance in bold

In our cohort, 259 patients were analysed. The SO group has 179 patients and Densiron group has 80 patients. As expected, there are several significant differences in baseline characteristics and outcomes of patients with different tamponade agents (Table 1). Similarly, there are significant differences between pre and postoperative visual outcomes by tamponade agent (Fig. 1).

**Table 1:** Baseline clinical and operative characteristics of primary retinal detachments

	Total	Silicone Oil	Densiron	**p Value**
Total	259	179	80	-
Baseline characteristics				
Age (years, IQR)	61 (49 to 71)	61 (49 to 70)	60 (49 to 73)	0.829
Gender (% Male)	187 (72.2%)	131 (73.2%)	56 (70.0%)	0.653
Laterality (% Right)	137 (52.9%)	92 (51.4%)	45 (56.3%)	0.502
Ocular Co-morbidities	97 (37.5%)	72 (40.2%)	25 (31.3%)	0.211
High Myope (% Yes)	22 (8.5%)	13 (7.3%)	9 (11.3%)	0.336
Glaucoma (% Yes)	8 (3.1%)	3 (1.7%)	5 (6.3%)	0.112
Preoperative lens*				
Phakic	142 (62.6%)	101 (63.9%)	41 (59.4%)	0.680
Pseudophakic	78 (34.4%)	53 (33.5%)	25 (36.2%)	
Aphakic	7 (3.1%)	4 (2.5%)	3 (4.3%)	
Macula status				
Off	201 (78.8%)	147 (83.5%)	54 (68.4%)	**0.008**
On	54 (21.2%)	29 (16.5%)	25 (31.6%)	
Giant retinal tear	11 (4.2%)	8 (4.5%)	3 (3.8%)	1.000
PVR C	65 (25.1%)	54 (30.2%)	11 (13.8%)	**0.005**
Pre-operative VA (logMAR)	1.50 (0.60 to 2.40)	1.60 (0.60 to 2.40)	0.80 (0.35 to 1.85)	** < 0.001**
Surgical Characteristics				
Retinectomy Performed	22 (8.5%)	19 (10.6%)	3 (3.8%)	0.090
Perfluorocarbon used	64 (24.7%)	53 (29.6%)	11 (13.8%)	**0.008**
Combined Buckle/PPV	19 (7.3%)	19 (10.6%)	0 (0.0%)	**0.001**
Combined Phacovitrectomy	19 (7.3%)	15 (8.4%)	4 (5.0%)	0.443
Epiretinal Membrane Peeled	2 (0.8%)	2 (1.1%)	0 (0.0%)	1.000
Retinopexy				
Cryotherapy only	33 (12.9%)	16 (9.1%)	17 (21.5%)	**0.009**
Endolaser only	139 (54.5%)	105 (59.7%)	34 (43.0%)	**0.015**
Cryotherapy and endolaser	83 (32.5%)	55 (31.3%)	28 (35.4%)	0.564
Three hundred and sixty laser	120 (46.3%)	87 (48.6%)	33 (41.3%)	0.284

Age is reported as median (interquartile range) and Kruskal Wallis test used to compare continuous variables

Chi Squared test to compare more than two nominal groups

Statistical significance in bold

*PVR *Proliferative vitreoretinopathy

^*^Pre-operative lens status could not be determined in 32 patients

Compared to Densiron, the SO group had higher proportion of macular off retinal detachment (p = 0.008), higher rates of PVR C (p = 0.005), lower pre-operative VA (p < 0.001), and higher rates of combined scleral buckle and PPV (p = 0.001). However, baseline characteristics such as age, gender, high myopia, glaucoma rate, and ocular co-morbidities were similar between both groups (Table 1).

To enable risk adjusted comparisons; various multivariable regression models were conducted.

### Primary outcome

Table 2 includes a multivariable linear regression models for logMAR gain following primary RRD repair, including only pseudophakic patients to reduce the confounder of cataracts (n = 187). In this model, 187 patients (70.7%) were included, 127 (71.8%) and 60 (77.9%) of total SO and Densiron patients respectively. Low logMAR gain was found to be significantly associated with better pre-operative logMAR and a combined scleral buckle with vitrectomy with no difference detected between SO and Densiron tamponade.

**Table 2:** Multivariable linear regression model for logMAR gain following primary retinal detachment repair

Independent variable	B Coefficient (95% CI)	p Value
Demographics & baseline characteristics		
Age	0.002 (− 0.005 to 0.008)	0.613
Male Gender (vs Female)	0.095 (− 0.122 to 0.312)	0.390
Ocular Comorbidities	− 0.166 (− 0.395 to 0.063)	0.154
High Myopia	− 0.029 (− 0.386 to 0.328)	0.873
Macular Status = ON	0.052 (− 0.232 to 0.336)	0.716
Giant Retinal Tear	0.269 (− 0.249 to 0.786)	0.307
Pre-Op Visual Acuity (logMAR)	0.754 (0.616 to 0.893)	** < 0.001**
Proliferative Vitreoretinopathy C	0.030 (− 0.271 to 0.331)	0.845
Intraoperative characteristics		
Silicone oil tamponade (REF Densiron)	0.165 (− 0.057 to 0.388)	0.143
Perfluorocarbon	− 0.154 (− 0.404 to 0.095)	0.223
Retinectomy	− 0.506 (− 1.031 to 0.020)	0.059
Combined PPV / Buckle	− 0.651 (− 1.061 to − 0.241)	**0.002**
Combined phacovitrectomy	0.137 (− 0.390 to 0.664)	0.608
Three Sixty EndoLaser	− 0.021 (− 0.225 to 0.184)	0.843
Postoperative characteristics		
Duration of Oil	0.000 (− 0.001 to 0.001)	0.585

Only Pseudophakic patients at final visual acuity were included, n = 187

Significance defined as p < 0.05. Significant values in bold

A Low logMAR gain was found to be significantly associated with better pre-operative logMAR and combined scleral buckle with vitrectomy

### Secondary outcomes

On univariate analysis, SO had significantly higher rates of retinectomy than Densiron in patients that did not have initial retinectomy (p = 0.005).

**Fig. 2:**
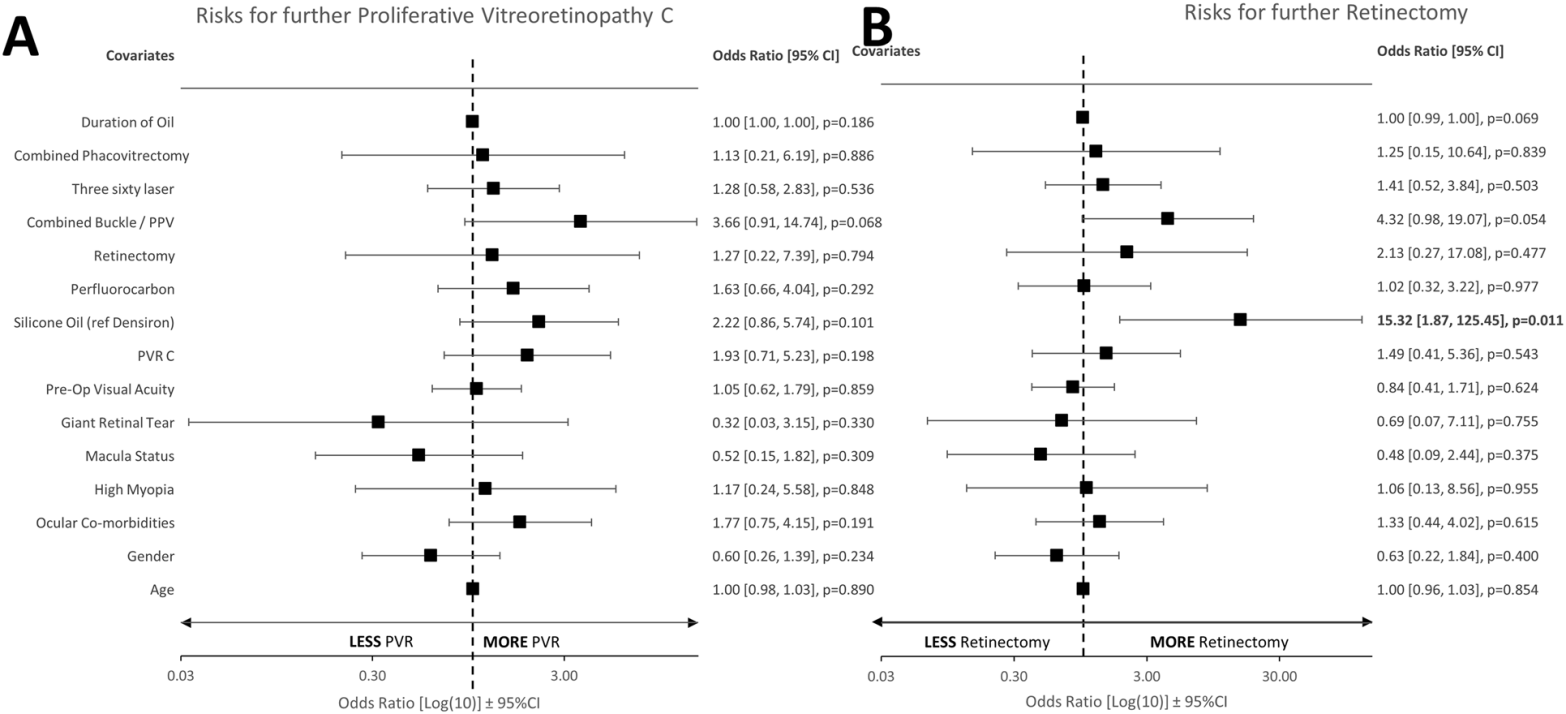
Forest Plots of Multivariable binary logistic regression model following primary retinal detachment repair. Significance defined as p < 0.05. Significant values in bold. **A** No significant risk factors were identified to increase risk for proliferative vitreoretinopathy formation (PVR). Combined Buckle / PPV had a had a trend toward significance (*p* = 0.068)*. ***B** Silicone oil relative to Densiron was significantly associated with increased retinectomy rate (*p* = 0.011). Combined buckle / PPV had a trend toward significance (*p* = 0.054)

Figure 2 includes multivariable binary logistic regression models for assessing risks for (A) further PVR C rate and (B) further retinectomy rate. SO had a trend but a non-significant increase in subsequent proliferative vitreoretinopathy C rate compared to Densiron, and no significant risk factors were identified. SO (compared to Densiron) was the only factor found to significantly increase subsequent retinectomy rate. Initial retinectomy was not found to be a significant risk factor for further retinectomy. Combined buckle with vitrectomy had a trend towards significance for further retinectomy rate.

The outcomes of primary RRD are found in Table 3. Densiron, compared to SO, had shorter duration of oil tamponade (p < 0.001) and had higher rates of successful ROSO (p = 0.030). We found no difference in subsequent RRD rate, or in rates of glaucoma surgery (for those without and with history of glaucoma pre-operatively, Table 3). If patients required subsequent RRD surgery, gas tamponade was more likely to be used for Densiron than SO tamponade (p = 0.004).

**Table 3:** Outcomes of primary retinal detachment surgery by oil type

	Total	Silicone Oil	Densiron	p Value
Total	259	179	80	
Postoperative Lens*				
Phakic	48 (18.9%)	34 (19.2%)	14 (18.2%)	0.307
Pseudophakic	187 (73.6%)	127 (71.8%)	60 (77.9%)	
Aphakic	16 (6.3%)	14 (7.9%)	2 (2.6%)	
Duration of oil (days)	133 (77 to 222)	163 (91 to 239)	85 (61 to 133)	** < 0.001**
Days Follow up	411 (207 to 729)	447 (237 to 765)	300 (178 to 675)	
Removal of tamponade (%)	193 (74.5%)	126 (70.4%)	67 (83.8%)	**0.030**
Primary Failure at six-months (%)	26 (10.0%)	18 (10.1%)	8 (10.0%)	1.000
Further RRD Surgery (% Yes)	75 (29.0%)	51 (28.5%)	24 (30.0%)	0.882
0	184 (71.0%)	128 (71.5%)	56 (70.0%)	0.844
1	53 (20.5%)	37 (20.7%)	16 (20.0%)	
≥ 2	22 (8.5%)	14 (7.8%)	8 (10.0%)	
Further PVR C (% Yes)	42 (16.2%)	33 (18.4%)	9 (11.3%)	0.201
No initial PVR C (% Yes)	26 (13.4%)	21 (16.8%)	5 (7.2%)	0.078
Initial PVR C (% Yes)	16 (24.6%)	12 (22.2%)	4 (36.4%)	0.442
Further Retinectomy rate	23 (8.9%)	22 (12.3%)	1 (1.3%)	**0.003**
No initial retinectomy (% Yes)	20 (8.4%)	19 (11.9%)	1 (1.3%)	**0.005**
Initial retinectomy (% Yes)	3 (13.6%)	3 (15.8%)	0 (0.0%)	1.000
Further ERM Surgery	12 (4.7%)	9 (5.1%)	3 (3.8%)	0.759
Required Glaucoma procedures (all)	9 (3.5%)	5 (2.8%)	4 (5.0%)	0.464
No History of Glaucoma	9 (3.5%)	5 (2.8%)	4 (5.2%)	0.459
Known Glaucoma Patient	0 (0.0%)	0 (0.0%)	0 (0.0%)	-
Required Glaucoma Surgery (tube/trabeculectomy/cyclodiode)	7 (2.7%)	4 (2.2%)	3 (3.8%)	0.680
Subsequent Tamponade				-
Oil	49 (65.3%)	34 (66.7%)	15 (62.5%)	0.797
Densiron	17 (22.7%)	14 (27.5%)	3 (12.5%)	0.237
Gas	18 (24.0%)	7 (13.7%)	11 (45.8%)	**0.004**
Post-Operative VA (logMAR)	0.80 (0.50 to 1.60)	1.00 (0.60 to 1.60)	0.50 (0.30 to 1.00)	** < 0.001**
LogMAR Gain	0.20 (− 0.30 to 0.80)	0.30 (− 0.30 to 0.90)	0.20 (− 0.25 to 0.65)	0.859

Age is reported as median (interquartile range) and Kruskal Wallis test used to compare continuous variables

Chi Squared test to compare more than two nominal groups

Statistical significance in bold

Postoperative lens status could not be determined in three patients

*GRT* Giant retinal tear, *PVR *proliferative vitreoretinopathy

## Discussion

This study is the largest comparative case series in the literature at the time of publication between SO and Densiron and is the only manuscript involving multivariable regression analyses comparisons.

Our data demonstrate that although there was no difference in logMAR gain between SO and Densiron in pseudophakic patients at final VA on risk adjusted multivariable linear regression analysis, there was a significantly higher retinectomy rate for SO on both univariate and binary logistic multivariable analyses. No difference in subsequent retinal detachment rate was found between the two tamponade agents. Our study also found no difference in subsequent PVR, glaucoma procedures, or ERM peels between the tamponade agents. Interestingly we found a trend toward significance for increased retinectomy rate in the combined scleral buckle / PPV group on multivariable regression. As a combined procedure should be retinectomy sparing, this suggests that inserting a buckle in SO cases does not reduce the risk of requiring a retinectomy.

Our results bring to light data which may reassure surgeons when considering alternatives to SO for patients. Wong et al. in 2009 had concluded that Densiron was associated with an elevated intraocular pressure in the early post-operative period. This difference was initially clinically significant up to day 14, however at week four, the intraocular pressure difference between the groups was no longer significant (P = 0.17) [8].

In a case series of 180 eyes in 2010, Romano et al. found that the use of Densiron as an endotamponade in PPV was not significantly associated with higher intraocular pressure [9]. Our study consolidates this finding clinically, as we did not find a difference in rate of glaucoma surgery between groups.

Semeraro et al. evaluated the inflammation associated with Densiron and standard silicone oil by measuring the aqueous IL-1a and prostaglandin-E2 levels. They concluded that Densiron caused a more severe inflammatory reaction in comparison [10]. Our cohort reflects a lack of clinical difference despite these findings, with no evidence that Densiron is more inflammatory than silicone oil, or that there is increased PVR or retinectomy rate.

Overall, although we report a primary success rate of 90.0% at six months, on longer follow up in median 411 (interquartile range] 207 to 729) days, we find 71.0% did not require subsequent RRD surgery at the final follow up visit, with no difference between both tamponades. In the literature there is a wide range of primary success using oil tamponade in primary RRD repair (63.0% to 87.6%) [1, 11–14], reflecting the heterogeneity between studies, in inclusion criteria, follow up duration and definition of primary failure.

### Study limitations and strengths

The limitations of our study include its retrospective nature and lack of case randomization including the location and extent of detachment. Despite this, our study has several strengths. A retrospective analysis allowed us to collate a large case series with adequate numbers in one unit to compare outcomes between SO and Densiron tamponade with risk adjusted multivariable regression analyses, to demonstrate its safety profile of each tamponade relative to each other.

## Conclusion

We report on our experience in using Densiron as a primary tamponade relative to SO. Despite reports of raised glaucoma, increased inflammation, and the risk of increased superior retinectomy compared to SO, we found significantly lower retinectomy rate and a trend towards significance for reduced PVR and better visual outcomes compared to SO. Although patients requiring glaucoma surgery was higher in Densiron, this was non-significant. We find that Densiron is as safe as SO for primary RD repair for visual outcomes, glaucoma surgery rate and PVR formation, albeit, with a lower retinectomy rate.

## Data Availability

Data are available upon reasonable request.

## References

[1] Kocak I, Koc H (2013). Comparison of Densiron 68 and 1000 cSt silicone oil in the management of rhegmatogenous retinal detachment with inferior breaks. Int J Ophthalmol.

[2] Caporossi T, Franco F, Finocchio L, Barca F, Giansanti F, Tartaro R (2019). Densiron 68 heavy silicone oil in the management of inferior retinal detachment recurrence: analysis on functional and anatomical outcomes and complications. Int J Ophthalmol.

[3] Hiscott P, Magee RM, Colthurst M, Lois N, Wong D (2001). Clinicopathological correlation of epiretinal membranes and posterior lens opacification following perfluorohexyloctane tamponade. Br J Ophthalmol.

[4] Li W, Zheng J, Zheng Q, Wu R, Wang X, Xu M (2010). Clinical complications of Densiron 68 intraocular tamponade for complicated retinal detachment. Eye.

[5] Heimann H, Stappler T, Wong D (2008). Heavy tamponade 1: a review of indications, use, and complications. Eye.

[6] Day AC, Donachie PHJ, Sparrow JM, Johnston RL (2015). The Royal College of Ophthalmologists’ National Ophthalmology Database study of cataract surgery: report 1, visual outcomes and complications. Eye (Basingstoke).

[7] Moussa G, Bassilious K, Mathews N (2021). A novel excel sheet conversion tool from Snellen fraction to LogMAR including ‘counting fingers’, ‘hand movement’, ‘light perception’ and ‘no light perception’ and focused review of literature of low visual acuity reference values. Acta Ophthalmol.

[8] Wong D, Kumar I, Quah SA, Ali H, Valdeperas X, Romano MR (2009). Comparison of postoperative intraocular pressure in patients with Densiron-68 vs conventional silicone oil: a case-control study. Eye.

[9] Romano MR, Angi M, Romano V, Parmeggiani F, Campa C, Valldeperas X (2010). Intraocular pressure changes following the use of silicone oil or Densiron® 68 as endotamponade in pars plana vitrectomy. Clin Ophthalmol.

[10] Semeraro F, Russo A, Morescalchi F, Gambicorti E, Vezzoli S, Parmeggiani F (2019). Comparative assessment of intraocular inflammation following standard or heavy silicone oil tamponade: a prospective study. Acta Ophthalmol.

[11] Herbrig E, Sandner D, Engelmann K (2007). Anatomical and functional results of endotamponade with heavy silicone oil—Densiron® 68—in complicated retinal detachment. Ophthalmic Res.

[12] Berker N, Batman C, Ozdamar Y, Eranil S, Aslan O, Zilelioglu O (2007). Long-term outcomes of heavy silicone oil tamponade for complicated retinal detachment. Eur J Ophthalmol.

[13] Joussen AM, Rizzo S, Kirchhof B, Schrage N, Li X, Lente C (2011). Heavy silicone oil versus standard silicone oil in as vitreous tamponade in inferior PVR (HSO Study): interim analysis. Acta Ophthalmol.

[14] Stappler T, Heimann H, Wong D, Gibran SK, Groenewald C, Pearce IA (2008). Heavy tamponade 2 Densiron 68® in routine clinical practice: anatomical and functional outcomes of a consecutive case series. Eye.

